# Draft genome sequence of the obligate bacterial endosymbiont *Buchnera aphidicola* of the aphid *Uroleucon formosanum*

**DOI:** 10.1128/mra.00449-25

**Published:** 2025-08-11

**Authors:** Hao Liu, Jingbo Li, Cailing Li, Liyun Jiang, Gexia Qiao, Jing Chen

**Affiliations:** 1 State Key Laboratory of Animal Biodiversity Conservation and Integrated Pest Management, Institute of Zoology, Chinese Academy of Sciences 53024, Beijing, China; 2 College of Life Sciences, University of Chinese Academy of Sciences, Beijing, China; University of Maryland School of Medicine, Baltimore, Maryland, USA

**Keywords:** insect endosymbiont, genome sequencing, phytotoxin tolerance

## Abstract

The genome of the obligate bacterial endosymbiont *Buchnera aphidicola* from the aphid *Uroleucon formosanum* was sequenced and assembled using Illumina paired-end sequencing. Genomic functional analysis revealed that *B. aphidicola* can assist its host in counteracting oxidative stress induced by plant-derived sesquiterpene lactones.

## ANNOUNCEMENT

The aphid *Uroleucon formosanum* (Takahashi, 1921) (Hemiptera: Aphididae) is a common herbivore on plants of the family Asteraceae in eastern Asia (1). It infests economically important crops, including lettuce (*Lactuca sativa*) and medicinal plants (2, 3), and can cause serious yield loss in lettuce (4). These plants usually contain sesquiterpene lactones that have negative effects on the growth and development of insects and increase their mortality (5, 6). The obligate endosymbiont of aphids, *Buchnera aphidicola*, can provide essential amino acids to help aphids overcome nutrient deficiency caused by phloem feeding (7). To date, *Buchnera* genomes are available for only two species of *Uroleucon*, *Uroleucon ambrosiae* (NC_017259) (8) and *Uroleucon sonchi* (NZ_CP047588). Here, we report the genome assembly of the *Buchnera* from *U. formosanum*.

**Fig 1:**
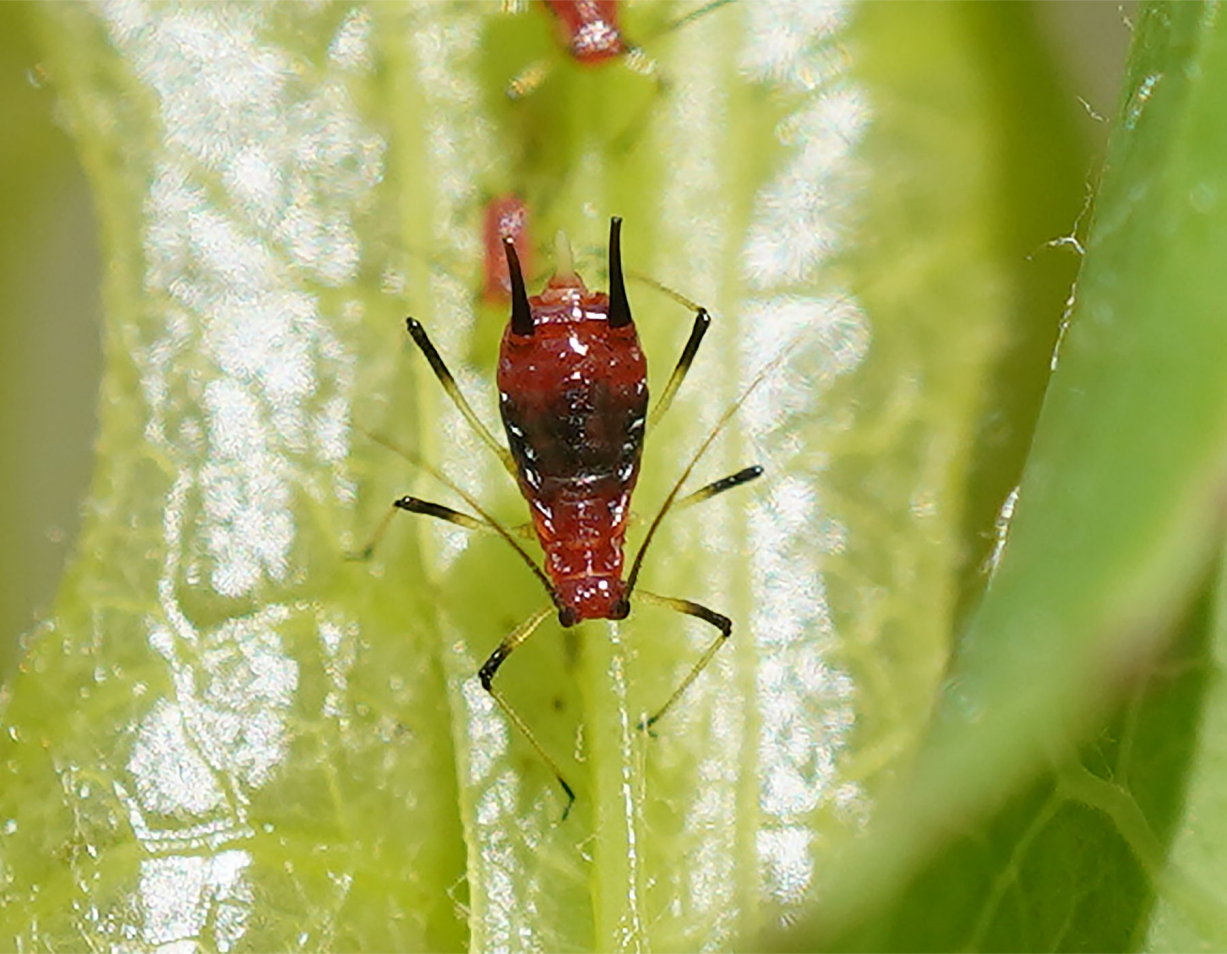
*Uroleucon formosanum* feeding on *Lactuca sativa* in the field.

The aphid samples (voucher number 52687, Fig. 1) were originally collected on *L. sativa* from Liu’an, Anhui, China (31.33°N, 116.37°E) in August 2021 and subsequently reared on lettuce plants in the laboratory of the Institute of Zoology, Chinese Academy of Sciences, Beijing, China. The cultivation conditions were controlled at a temperature of 24 ± 1°C, a relative humidity of 65% ± 5%, and a 16/8 h light/dark photoperiod. Genomic DNA was extracted from whole bodies of 15 apterous adult individuals using the cetyltrimethylammonium bromide (CTAB) method. DNA libraries were prepared using the VAHTS Universal Plus DNA Library Prep Kit for Illumina (Illumina, San Diego, CA, United States). Sequencing was then performed on an Illumina NovaSeq 6000 platform with a read length of 150 bp. Raw sequencing data were processed to remove adapters and filter low-quality reads, yielding 43,602,433 clean reads. These reads were used for *de novo* genome assembly with SPAdes v.3.15.5 (--plasmid --only-assembler) (9). The assembly was aligned to the reference genome, *B. aphidicola* (*U. ambrosiae*), using MUMmer v.3.1 (10). Matching scaffolds were extracted and examined manually for circularity, and overlapping end regions were trimmed to generate the draft genome. The genome was then polished with Pilon v.1.24 (11) and manually rotated to begin at the same position as the reference genome. The coverage of reads for the genome was calculated using BWA v.0.7.17 (12) and SAMtools v.1.15 (13). Assembly integrity was evaluated by CheckM v.1.2.1 (14). Genome annotation was conducted using NCBI PGAP v.6.4 (15). Functional analysis was performed with EggNOG-mapper v.2 (16). Default parameters were used for all software unless otherwise specified.

The final genome assembly of *Buchnera* (*U. formosanum*) includes one circular chromosome of 614,287 bp long with a GC content of 24.34% and one circular plasmid, pLeu, which is 7,793 bp long with a GC content of 25.60% (Table 1). The plasmid pTrp, which carries genes involved in tryptophan biosynthesis (17), was not successfully assembled. The CheckM assessment showed a high genome completeness score of 99.41%. A total of 586 genes were predicted, including 545 protein-coding genes, 3 pseudo genes, 32 tRNAs, 3 rRNAs, and 3 ncRNAs. Gene Ontology (GO) annotations show that the genes *trxA*, *trxB*, and *sodA* are involved in detoxification. The thioredoxin (*trxA*) and thioredoxin reductase (*trxB*) genes are key components of the thioredoxin system, which plays a critical role in cellular responses to changes in oxygen concentration and oxidative stress (18). The *sodA* gene encodes manganese superoxide dismutase, which catalyzes the conversion of superoxide radicals into hydrogen peroxide and oxygen, thereby mitigating oxidative stress (19, 20). Sesquiterpene lactones have been found to induce oxidative stress by depleting reduced glutathione and generating reactive oxygen species (ROS), which, at high levels, can be toxic and trigger apoptosis (21, 22). Therefore, the genome of *Buchnera* (*U. formosanum*) contains genes with the potential to counteract oxidative stress induced by plant-derived sesquiterpene lactones within the aphid host.

**TABLE 1:** Genomic features of *Buchnera aphidicola* (*Uroleucon formosanum*)

Genomic feature	Value
Total length (bp)	622,080
Chromosome length (bp)	614,287
Plasmid pLeu length (bp)	7,793
Genome coverage (chromosome)	681×
Genome coverage (pLeu)	878×
GC content (total) (%)	24.36
GC content (chromosome) (%)	24.34
GC content (plasmid pLeu) (%)	25.60
CheckM completeness (%)	99.41
CheckM contamination (%)	0
CheckM heterogeneity (%)	0
No. of genes	586
No. of protein-coding genes	545
No. of rRNAs	3
No. of tRNAs	32
No. of ncRNAs	3
No. of pseudo genes	3

## Data Availability

The genome sequences of *Buchnera aphidicola* have been deposited in GenBank under the accession numbers CP187398 and CP187399. The BioSample and BioProject accession numbers are SAMN47576917 and PRJNA1242035, respectively. Raw Illumina sequencing data have been deposited in the NCBI Sequence Read Archive (SRA) database under the accession number SRR32922889.
